# A picture of health: determining the core population served by an urban NHS hospital trust and understanding the key health needs

**DOI:** 10.1186/s12889-021-12373-5

**Published:** 2022-01-12

**Authors:** Thomas Beaney, Jonathan M. Clarke, Emily Grundy, Sophie Coronini-Cronberg

**Affiliations:** 1 Department of Primary Care & Public Health, School of Public Health, Imperial College London, London, W6 8RP UK; 2 NIHR Applied Research Collaboration (ARC) Northwest London, 4th Floor, Chelsea and Westminster Hospital, 369 Fulham Road, London, SW10 9NH UK; 3 Centre for Mathematics of Precision Healthcare, Department of Mathematics, Huxley Building, South Kensington Campus, Imperial College London, London, SW7 2AZ UK; 4 Chelsea and Westminster Hospital NHS Foundation Trust, 369 Fulham Road, London, SW10 9NH UK

**Keywords:** Hospital catchments, Healthcare public health, Health needs assessment

## Abstract

**Background:**

NHS hospitals do not have clearly defined geographic populations to whom they provide care, with patients able to attend any hospital. Identifying a core population for a hospital trust, particularly those in urban areas where there are multiple providers and high population churn, is essential to understanding local key health needs especially given the move to integrated care systems. This can enable effective planning and delivery of preventive interventions and community engagement, rather than simply treating those presenting to services. In this article we describe a practical method for identifying a hospital’s catchment population based on where potential patients are most likely to reside, and describe that population’s size, demographic and social profile, and the key health needs.

**Methods:**

A 30% proportional flow method was used to identify a catchment population using an acute hospital trust in West London as an example. Records of all hospital attendances between 1^st^ April 2017 and 31^st^ March 2018 were analysed using Hospital Episode Statistics. Any Lower Layer Super Output Areas where 30% or more of residents who attended any hospital for care did so at the example trust were assigned to the catchment area. Publicly available local and national datasets were then applied to identify and describe the population’s key health needs.

**Results:**

A catchment comprising 617,709 people, of an equal gender-split (50.4% male) and predominantly working age (15 to 64 years) population was identified. Thirty nine point six percent of residents identified as being from Black and Minority Ethnic (BAME) groups, a similar proportion that reported being born abroad, with over 85 languages spoken. Health indicators were estimated, including: a healthy life expectancy difference of over twenty years; bowel cancer screening coverage of 48.8%; chlamydia diagnosis rates of 2,136 per 100,000; prevalence of visible dental decay among five-year-olds of 27.9%.

**Conclusions:**

We define a blueprint by which a catchment can be defined for a hospital trust and demonstrate the value a hospital-view of the local population could provide in understanding local health needs and enabling population-level health improvement interventions. While an individual approach allows tailoring to local context and need, there could be an efficiency saving were such public health information made routinely and regularly available for every NHS hospital.

**Supplementary Information:**

The online version contains supplementary material available at 10.1186/s12889-021-12373-5.

## Background

To date, analysis of National Health Service (NHS) healthcare activity in England has primarily focussed on commissioning organisations that fund healthcare for their designated populations. Their constituent population is bound by membership rules and therefore comparatively easy to define. Whereas in primary care, the duty of care extends only to registered patients, in secondary care, access is universal through the Emergency Department, and initiatives such as Choose and Book [1].

Using data only on those attending hospital services fails to account for those groups not seeking care and does not tell us whether these individuals are unable to access care, their needs are being managed elsewhere, or they are healthy [2, 3]. Understanding the ‘effective’ population is critically important as it provides a baseline denominator from which to evaluate service provision, unmet need and inequity of access.

The need for such modelling is imperative as the NHS moves towards developing an integrated care system (ICS) focussed on population health improvement [4]. This will see pooled risks and budgets to deliver services which incentivise keeping people well, regardless of where they access services. This contrasts with the existing fee-for-service system [4].

There have been previous attempts to define hospital catchments that represent the population served by a hospital though these approaches tend to generate mutually exclusive areas [3, 5]. This is problematic in urban areas where there tend to be several commissioning and provider organisations, coupled with a more mobile population, meaning hospital choice may change for different services or change with waiting list times for appointments [6].

A range of different methods may be used to define a catchment population. In some contexts, a catchment population may not need to correspond to a delineated geographic area and may be defined based on the stratified proportional allocation of patients attending the hospital trust from across the entire country [3]. Where the aim of a catchment is to produce a defined geographic area, methods involve the allocation of an administrative geographic area and its constituent population to the catchment. Two commonly used approaches are the ‘First Past the Post’ (FPTP) method, which assigns  a geographic unit to the single trust providing care to the highest proportion of patients within the geographic unit and the Proportional Flow (PF) method, where geographic units are assigned to a trust if the proportion of all patients resident within the geographic unit attending a trust exceeds a defined threshold [7]. The advantage of the PF method is that the threshold remains constant, whereas with FPTP, the threshold for assignment will change according to the number of providers, which may be more of a concern in urban areas [7].

This study applied PF methodology to an acute trust located in West London. Chelsea and Westminster Hospital NHS Foundation Trust (CWFT) comprises two hospitals: West Middlesex University Hospital, and Chelsea and Westminster Hospital. The trust offers full maternity, emergency and children’s services, some specialised services (e.g. burns), and community clinics (e.g. sexual health)*.* Patients attending CWFT predominantly live in one of seven local authorities (LAs): Ealing, Hammersmith & Fulham, Hounslow, Kensington & Chelsea, Richmond-upon-Thames, Wandsworth, and Westminster, a combined 253 square kilometre area, with 1.7 million registered residents [8]. This area also covers seven Clinical Commissioning Groups (CCGs), whose boundaries are not necessarily co-terminus with the above LA ones; falls across two ICSs (North West London and South West London) [9]; contains seven general hospitals, plus stand-alone specialist cancer, pulmonary, ophthalmology and mental health service providers.

This complex health eco-system underscores the challenges of defining a denominator population agnostic to geographical boundaries that takes the underlying population structure into account. This study aims to use administrative healthcare data to define a single geographic catchment area of a hospital trust using the Proportional Flow method. Using CWFT as an example, we illustrate how knowledge of the population resident within a catchment area can be used by hospital trusts to describe a range of population health needs.

## Methods

Hospital Episode Statistics (HES) is a routinely collected dataset of all emergency department (A&E) attendances, admissions and outpatient appointments at all NHS hospitals in England [10]. Records of all hospital attendances to any trust by patients resident in England were extracted for the period between 1^st^ April 2017 and 31^st^ March 2018, inclusive. Hospital trusts were identified from the Estates Returns Information Collection [11]. For A&E attendances, only those to a consultant-led A&E service (AEDEPTTYPE=1) were selected, as some urgent care and walk-in centres are run by separate community providers, which in turn may run several such services across different locations which cannot be consistently disaggregated.

Lower-layer Super Output Area (LSOA) is a geospatial unit used to facilitate the reporting of small area statistics. LSOAs were primarily designed for the Census and are designed to be fairly homogenous in terms of population size to allow comparisons over time, or between areas. There are 32,844 LSOAs in England, with a minimum population of 1,000, and a mean of 1,700 [12].

### Patient allocation to trusts

For each LSOA, patients were identified and hospital attendance counts aggregated. Where individuals were associated with more than one LSOA over the year, the LSOA associated with the majority of attendances was assigned. In the case of ties, the LSOA was randomly allocated. Similarly, where individuals had presented to more than one hospital, the patient was assigned to the trust they attended most frequently and in the case of ties, were randomly allocated. The count of patients from each LSOA attending each trust was then divided by the total number of patients from the same area attending any trust. Proportions under 10% were suppressed and set to 0% for the purposes of data visualisation.

### Constructing the catchment population

A PF approach was chosen as this allows for cross-boundary patient movement to be captured and was felt to be more realistic particularly in an urban area where population movement is high and there are multiple providers. The threshold was chosen following a sensitivity analysis considering multiple thresholds ranging from 10 to 50%. The purpose was to minimise LSOAs either not being allocated at all, or being allocated to multiple trust catchments (Additional file 1, Fig. A1). Based on this analysis, an LSOA was assigned to the catchment area if at least 30% of patients residing in it attended that trust as their most frequently attended trust.

The ‘overall’ catchment (generated using A&E attendances, admissions and outpatient appointments) was compared to three catchments based on activity subsets: adult A&E; paediatric A&E; and maternity admissions (Additional file 1, Table A1). The overall catchment area representing all activity was selected for further description.

### Catchment population characteristics

The 2019 Office for National Statistics (ONS) estimates of the registered mid-year population of each LSOA in England was applied to generate an overall population sum [13]. The age and gender structure was calculated using age-specific banded LSOA-level population estimates. The distribution of deprivation was quantified using LSOA-level Index of Multiple Deprivation (IMD) decile data for England for 2019 [14]. The IMD score provides a composite data measure for each LSOA in England, and a rank relative to all other LSOAs. A score of 1 signifies the most deprived and 32,844 the least deprived LSOA.

### Ethnicity estimates

ONS publish population ethnicity estimates following each population Census. There is no nationally agreed method by which to create future projections of population ethnicity [15]. The Greater London Authority (GLA) produces projections of ethnicity by gender and single-year age band. Although derived from Census data, the figures are adjusted for variables including ethnicity, age-specific fertility rates, migration and are generally deemed robust [16, 17]. The projections reflect the rapidly changing ethnic profile, and high population churn experienced across London.

To determine the ethnic breakdown of the population, GLA LA-level housing-led age and gender-specific ethnicity population projections were applied to each of constituent LSOA falling within the catchment, and these estimates then summed to determine the broad overall ethnicity profile (White British/Irish; White Other; Black; Asian; Mixed; Other) [18].

### Estimates of health need

A range of open-access epidemiological data was applied to examine health needs. These datasets were available at number of different levels of geographical granularity (Table 2):

- LSOA-level data (e.g. children living in low income households): these datasets could be directly mapped onto the individual LSOA’s within the CWFT catchment area.
- Middle Layer Super Output Area (MSOA)-level data (e.g. healthy life expectancy): although larger than LSOAs, can still be reasonably be mapped to LSOAs.
- LA or CCG-level data (e.g. Measles, Mumps, & Rubella vaccination uptake): each LSOA was assigned to its parent LA or CCG, and the summary statistic (e.g. prevalence) then assigned to the population of each constituent LSOA falling with the catchment. This resulting numerator could then be summed across all constituent LSOAs and used with the total population estimate to derive an overall summary statistic.
- National data (e.g. alcohol consumption, disability prevalence): either where lower-level geographic data was not available, or inappropriate to use, national age- and gender-specific data were applied to the catchment population.

Applying LA or CCG-level data could mask any potential variation between LSOAs. Where national data was available disaggregated by IMD decile, an adjustment was performed to account for the relative distribution of deprivation within the LA or CCG (Additional file 1, p5). Existing ONS mapping of LSOAs to CCGs was used to assign constituent catchment LSOAs to their relevant local CCG [19]. Where epidemiological data related to a particular age-range, they were applied to the corresponding subset of the catchment. Where age-bands did not directly align and alternative bandings needed to be created, it was assumed the number of residents was equally distributed within each band. It was not always appropriate to use the catchment population as the denominator: five indicators used alternative, more relevant figures (Additional file 1, Table A2).

All methods were carried out in accordance with relevant guidelines and regulations. Python version 3.6.8, Pandas version 0.24 was used for data manipulation and analysis, and Folium version 0.9.0 for data visualisation. Application of epidemiological data was undertaken in Microsoft Excel 2010.

## Results

**Fig. 1:**
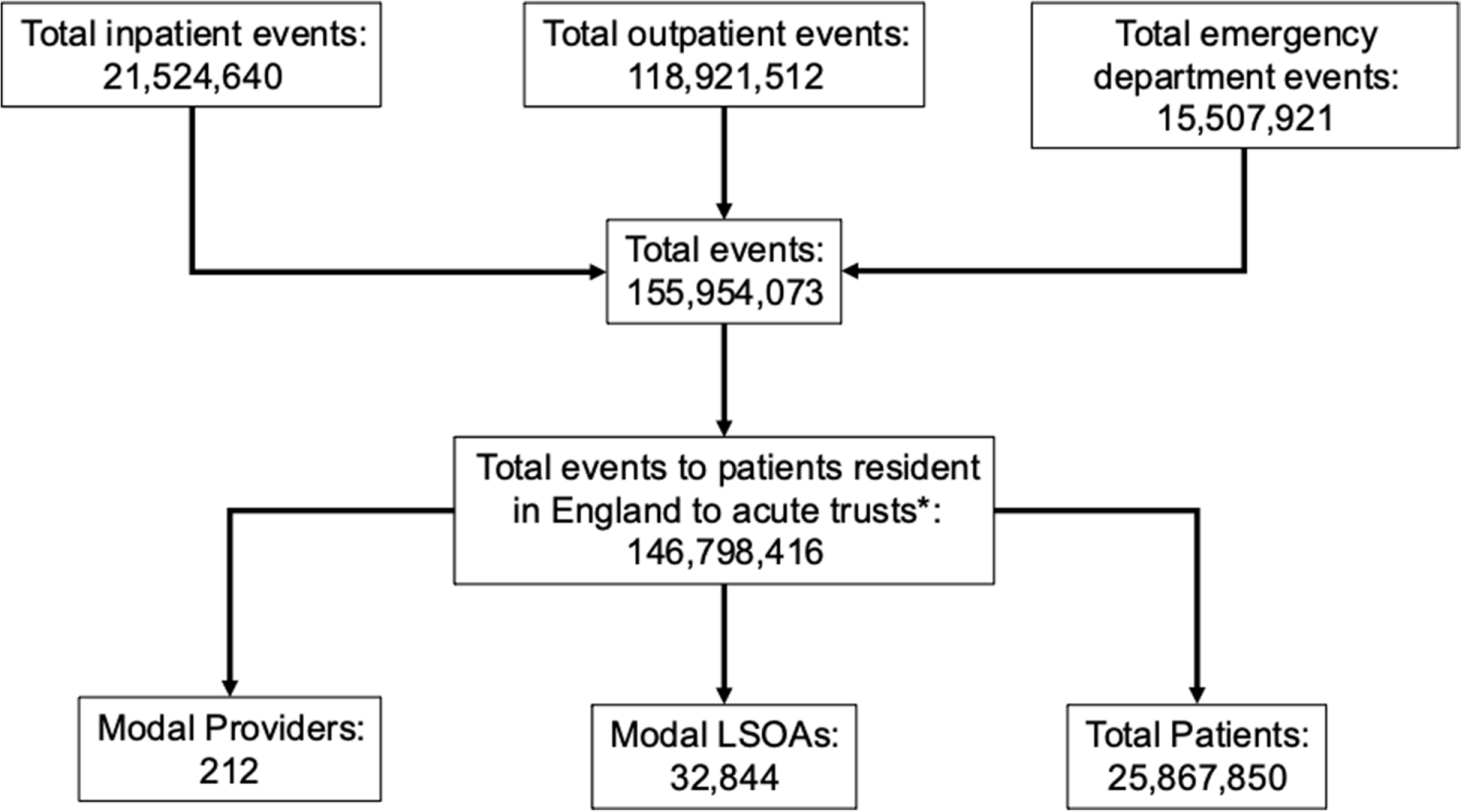
Flowchart showing derivation of Hospital Episode Statistics data. Footnote: acute trusts were defined as those whose 3-digit provider codes began with ‘R’

One hundred forty-six million seven hundred ninety-eight thousand four hundred sixteen events were identified, corresponding to 25,867,850 unique patients (Fig. 1). All 32,844 LSOAs and 212 hospitals in England were represented.

**Fig. 2:**
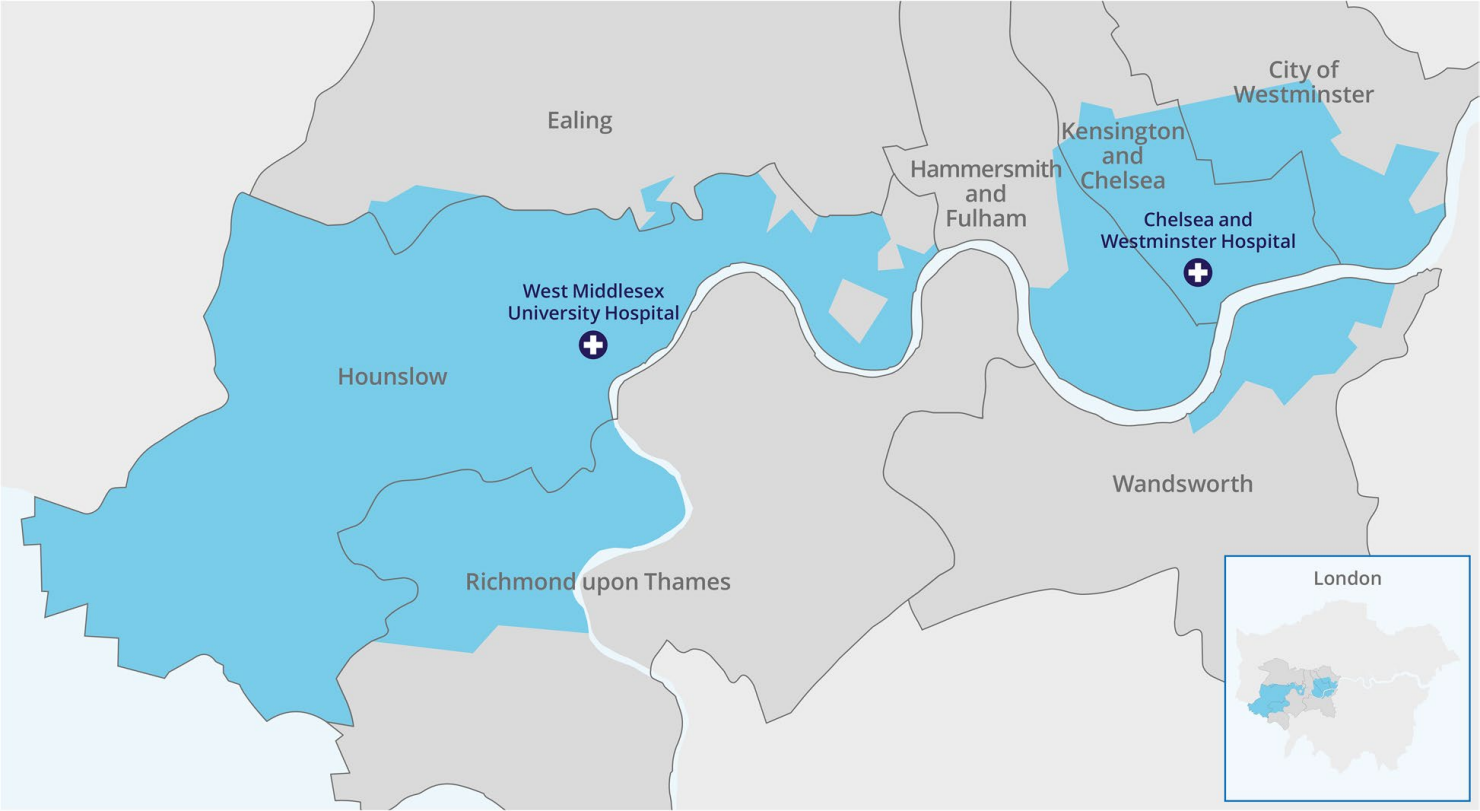
Map of the Chelsea and Westminster Hospital NHS Foundation Trust catchment area using a 30% proportional flow method. The inset panel shows the location of the catchment within Greater London

An overall catchment population of 617,709 people, drawn from 355 out of a total of 976 LSOAs across the seven surrounding boroughs, was generated for CWFT (Table 1). The proportion of patients from each LSOA assigned to CWFT ranged from 30.1 to 76.5% (Appendix Fig. A1). No LA area was entirely contained within the catchment (Fig. 2), and 682 LSOAs (2.1%) were identified with at least 10% of patients attending CWFT. The median proportion of local authority LSOAs contributing to the trust’s catchment was a third (33.0%), ranging from 2.0% (Ealing) to 95.1% (Hounslow).

**Table 1:** Derivation of catchment population of Chelsea and Westminster NHS Foundation Trust (CWFT) by local authority

	Local Authority (LA)	ICS region	LSOAs in CWFT catchment (N)	CWFT catchment population (N)	Proportion of CWFT catchment in LA (%)	Total LA population (N)	Proportion of LA population falling in CWFT catchment (%)
**Overall catchment population**	Ealing	NWL	4	6,622	1.1%	341,806	1.9%
	Hammersmith and Fulham	NWL	59	93,242	15.1%	185,143	50.4%
	Hounslow	NWL	135	257,860	41.7%	271,523	95.0%
	Kensington and Chelsea	NWL	70	106,992	17.3%	156,129	68.5%
	Richmond-upon-Thames	SWL	38	65,157	10.5%	198,019	32.9%
	Wandsworth	SWL	18	32,920	5.3%	329,677	10.0%
	Westminster	NWL	31	54,916	8.9%	261,317	21.0%
	**GRAND TOTAL**	**…**	**355**	**617,709**	**…**	**1,743,614**	**…**

LA total population estimates based on ONS Estimates of the population for the UK, England and Wales, Scotland and Northern Ireland: Mid-2019: April 2019 local authority district codes

*LSOA* Lower Super Output Area, *ICS* Integrated Care System, *NWL* North West London, *SWL* South West London

The single largest contributor to the catchment was Hounslow (41.7%), followed by Kensington and Chelsea (17.3%). The number of people from each LA contained in the catchment ranged from 6,622 (Ealing) to 257,860 (Hounslow). A positive skew was noted with a mean and median of 88,244 and 65,157 people, respectively. One in six (15.9%) people was from the South West London ICS. Corresponding catchment areas were also constructed, separately, representing adult A&E attendances, paediatric A&E attendances and maternity admissions, which demonstrated larger total populations (Additional file 1, Table A1). Further description of the trust catchment was applied to the overall catchment, representing all activity.

### Demographic features

**Fig. 3:**
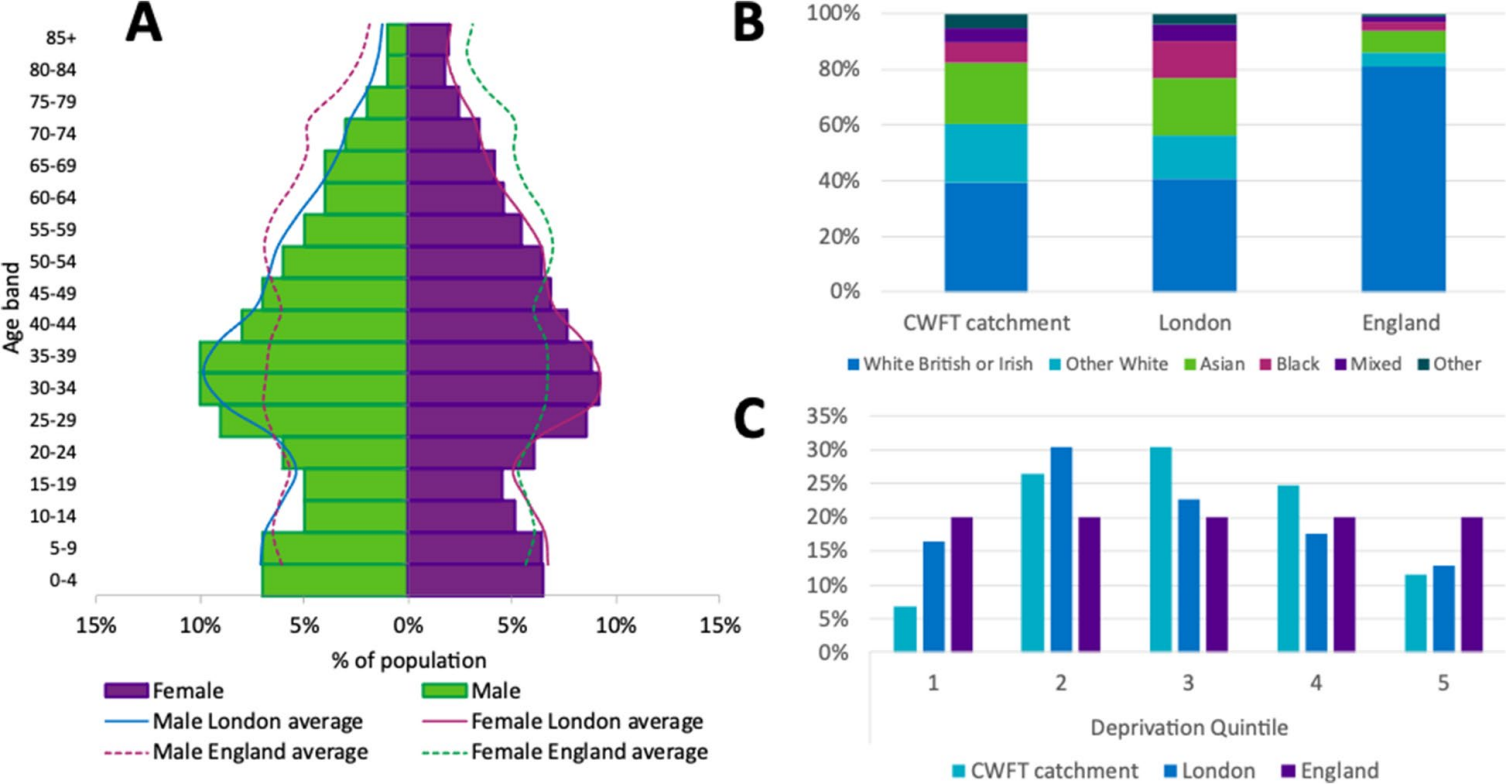
Summary of key demographic indicators for the CWFT catchment population with data for London and England as comparators. **A** Population pyramid for the CWFT ‘all activity’ catchment by gender compared to London and England. **B** Breakdown of populations of England, London and CWFT ‘all activity’ catchment by broad ethnic category. **C** Distribution of constituent LSOAs of CWFT ‘all activity’ catchment, London and England, by IMD 2019 quintile. Ethnicity of CWFT and London based on GLA estimates [18], England data based on ONS 2011 Census data [15]

We observed a roughly equal gender-split (50.4% male), predominantly (69.2%) working age (15 to 64 years) population with a median age of 36 years (Fig. 3). The total dependency ratio (ratio of <15 years and >64 years to those of working age) was 449 per 1,000, compared to 538 per 1,000 across England.

Two in five (39.6%) residents identify as being from Black and Minority Ethnic (BAME) groups, and 1 in 5 (21.1%) from white backgrounds other than ‘white British’ or ‘Irish’. Two in 5 people were born outside the United Kingdom or Ireland, with most common countries of birth including India (5.4%), Poland (2.4%), USA (2.0%) and France (2.0%). More than 87 languages are spoken locally with French, Polish and Punjabi the most common with over 10,000 speakers each, but many languages have fewer than 1,000 speakers. 6.8% of the population live in one of the 20% most deprived areas in the country.

### Estimate of health need

We found significant variation in life expectancy and healthy life expectancy between some of the most deprived and most affluent areas within the catchment. Among women, the maximum difference in life expectancy was 10.5 years, and up to 21.2 fewer years in good health, while for men these figures were 16.0 and 23.3 years, respectively.

Bowel cancer screening coverage amongst 60-64 year olds was estimated to be 48.8% within the catchment, lower than the national rate (60.1%). Chlamydia diagnosis rates amongst 15-24 year olds were estimated to be lower (2,136 per 100,000, compared to 2,610 per 100,000 across London, though both were higher than the national rate (1,975 per 100,000), while prevalence of visible dental decay among five-year-olds is higher (27.9%; London 27.0%; England 23.4%). Table 2 summarises all applied datasets and the resulting summary statistics.

**Table 2:** Health datasets applied to the CWFT ‘all activity’ catchment population showing the summary statistic, comparators with London and England and organised according to the geographical granularity of the dataset

Indicator name	Numerator	Denominator	Summary statistic	London	England	Date	Source
**LSOA**							
Children living in in low income households (children under 16 years)	16,480	118,290	13.9%	18.8%	17.0%	2016	GOV.UK Personal tax credits: Children in low-income families local measure: 2016 (snapshot as at 31 August 2016) https://www.gov.uk/government/statistics/personal-tax-credits-children-in-low-income-families-local-measure-2016-snapshot-as-at-31-august-2016
Fuel Poverty: Proportion of households estimated to be fuel poor	29,780 (households)	260,826 (households)	11.4%	11.8%	10.9%	2017	GOV.UK Fuel poverty sub-regional statistics https://www.gov.uk/government/statistics/sub-regional-fuel-poverty-data-2019
Overcrowding: Proportion of households experiencing overcrowding	24,839 (households)	252,531 (households)	9.8%	11.6%	4.8%	2011	ONS 2011 Census (NOMIS) QS412EW - Occupancy rating (bedrooms) https://www.nomisweb.co.uk/census/2011/qs412ew
**MSOA**							
Healthy life expectancy	n/a	n/a	**Females** Minimum: 57.1 years Maximum: 78.3 years **Males** Minimum: 56.9 years Maximum: 80.2	**Females** 51.3 – 78.3 **Males** 52.1 – 80.2	**Females** 46.1 – 78.3 **Males** 46.3 – 80.2	2009-13	PHE Fingertips – Local Health Profile https://fingertips.phe.org.uk/profile/local-health
Life expectancy	n/a	n/a	**Females** Minimum: 80.7 Maximum: 91.2 **Males** Minimum: 76.1 Maximum: 92.1	**Females** 76.1 – 94.2 **Males** 72.1 – 92.1	**Females** 72.3 – 100.3 **Males** 64.7 – 92.1	2013-17	PHE Fingertips – Local Health Profile https://fingertips.phe.org.uk/profile/local-health
**LA**							
Bowel cancer screening coverage (60-74 year olds)	34,738	71,121	48.8%	51.5%	60.1%	2019	PHE Fingertips – Public Health Outcomes Framework https://fingertips.phe.org.uk/profile/public-health-outcomes-framework
Chlamydia diagnoses (15-24yr olds)	1,437	67,245	2,136 per 100,000	2,610 per 100,000	1,975 per 100,000	2018	PHE Fingertips – Sexual and Reproductive Health Profile https://fingertips.phe.org.uk/profile/sexualhealth
Visible dental decay at 5 years	2,227	7,975	27.9%	27.0%	23.4%	2019/20	PHE Fingertips – Child and Maternal Health profile https://fingertips.phe.org.uk/profile/child-health-profiles
Estimated number of deaths attributable to air pollution (30+years)	211 deaths	3,232 deaths	6.5%	6.6%	5.2%	2018	PHE Fingertips – Public Health Outcomes Framework https://fingertips.phe.org.uk/profile/public-health-outcomes-framework
Excess weight: Prevalence of overweight (including obesity) at year 6 (10-11year olds)	4,491	12,790	35.1%	37.9%	34.3%	2018/19	PHE Fingertips – Public Health Outcomes Framework https://fingertips.phe.org.uk/profile/public-health-outcomes-framework
Uptake of free school meals (4-15 years)	12,926	85,781	15.1%	15.6%	13.5%	2018	PHE Fingertips - Child and Maternal Health profile https://fingertips.phe.org.uk/profile/child-health-profiles
MMR uptake - 2 doses (5 years)	5,943	7,975	74.5%	76.3%	86.4%	2018/19	PHE Fingertips - Child and Maternal Health profile https://fingertips.phe.org.uk/profile/child-health-profiles
Physical inactivity (19+ years)	98,982	482,010	20.5%	22.1%	21.4%	2018/19	PHE Fingertips – Physical Activity profile https://fingertips.phe.org.uk/profile/physical-activity
Recorded dementia prevalence (65+ years)	3,277	78,011	4.2%	4.5%	4.3%	2019	PHE Fingertips – Productive Healthy Aging profile https://fingertips.phe.org.uk/profile/healthy-ageing
School readiness – good level of development (aged 5)	5,900	7,975	74.0%	74.1%	71.8%	2018/19	PHE Fingertips – Public Health Outcomes Framework https://fingertips.phe.org.uk/profile/public-health-outcomes-framework
Smoking prevalence (18+ years) (APS)	60,629	487,813	12.4%	13.9%	14.4%	2018	PHE Fingertips - Local Tobacco Control Profiles https://fingertips.phe.org.uk/profile/tobacco-control
**CCG**							
Estimated common mental health disorders (16+ years)	91,050	499,419	18.2%	-	16.9%	2017	PHE Fingertips – Productive Healthy Aging profile https://fingertips.phe.org.uk/profile/healthy-ageing
**England**							
Bowel cancer incidence (10+ years)	301.68	537,197	56.16 per 100,000	-	67.3 per 100,000	2015-17 average	Cancer Research UK - Bowel cancer incidence statistics https://www.cancerresearchuk.org/health-professional/cancer-statistics/statistics-by-cancer-type/bowel-cancer/incidence#heading-One
Diagnosed and undiagnosed diabetes (16+ years)	36,135	499,419	7.2%	-	8.6%	2015	PHE - Analysis of estimates of diabetes prevalence across England 2015 https://assets.publishing.service.gov.uk/government/uploads/system/uploads/attachment_data/file/612306/Diabetesprevalencemodelbriefing.pdf
Disability prevalence (all ages)	117,290	617,709	19.0%	15.0%	21.0%	2014/15 – 2016/17 average	GOV.UK - Family Resources Survey: financial year 2016/17 – disability data tables https://www.gov.uk/government/statistics/family-resources-survey-financial-year-201617
Proportion of adults drinking above CMO recommended limit (16+ years)	83,543	394,099	21.2%	-	22.2%	2018	Health Survey for England 2018 – Adults’ health-related behaviours data tables (version 2) https://digital.nhs.uk/data-and-information/publications/statistical/health-survey-for-england/2018/health-survey-for-england-2018-data-tables

Some health indicators were similar to regional and national averages. For example, 35.1% children aged 10-11 years old in the catchment were estimated to be overweight or obese, compared to 37.9% in London and 34.4% in England. Similarly, 11.4% of households experienced fuel poverty, comparable to London (11.8%) and England (10.9%).

## Discussion

Using PF methodology, we have generated a core ‘catchment population’ for an urban hospital trust in England. This catchment identifies the geographic area over which people seeking care are likely to attend the trust, allowing us to describe the population’s size, demographics and social profile.

The catchment encompasses 620,000 people, equating to 1 in 14 London residents. It covers only parts of seven local authority areas, suggesting existing boundaries do not map across to trust activity: despite Chelsea and Westminster Hospital being located in the Royal Borough of Kensington and Chelsea, only two thirds (68.0%) of the borough’s LSOAs fall into the catchment. Instead, the River Thames appears to be a significant boundary in the south west of the catchment, though not for north east Wandsworth residents, an observation perhaps explained by the proximity of three bridges connecting north and south banks within a mile of each other.

There is also an apparent inverse relationship between distance people live from the trust and the proportion falling into the catchment, although with a notable exception in Hammersmith, which may be explained by the location of Charing Cross Hospital, which forms part of a different NHS hospital trust. This also may explain why the catchment appears as two geographically distinct regions (Fig. 2) though at lower thresholds the CWFT catchment area becomes a single unified geographic area (Appendix Fig. A1). Despite this area not being contiguous at the 30% threshold chosen, it should be regarded as a single catchment population owing to CWFT being a single organisation which shares a number of key clinical services between the two hospital sites.

Applying nationally-available data starts to tell the story about the community the trust serves and helps identify health needs. For example, the catchment’s age structure is younger than the England average: two-thirds (69.2%) are of working age, suggesting the expected unemployment surge in the wake of COVID-19 may hit this population very hard. It is well-documented that unemployment puts health at risk [20].

Joint Strategic Needs Assessments are statutory reviews of the current and future health and social care needs of LA residents [21]. Unlike for local government, there is no statutory duty for NHS hospitals to publish needs assessments. Our work illustrates that the catchment of an urban trust may not cover its constituent LA population equally, and by implication therefore may not reflect the health needs of their residents. Important nuances could be missed by only relying on LA data views and so deriving health indicators for a hospital’s population provides a novel opportunity to identify specific local needs. Chlamydia diagnosis rates, bowel cancer screening coverage, and dental decay rates are examples where the trust’s catchment population was found to diverge from regional and/or national comparators.

Understanding the unique characteristics of its local population allows an acute trust to prioritise addressing the most pressing health needs, engage proactively with prevention initiatives and, in our opinion, this data view opens up opportunities for transformative change. For example, CWFT’s catchment population was found to be ethnically very diverse: 40% identify as being from BAME groups, compared to 14% nationally, and a higher proportion of ‘white other’ groups compared to either London or England averages. Having baseline data provides a denominator for health equity audits, for example comparing the ethnic composition of hospital patients, staff or even governance boards to that of the local community.

### Preventive outreach

While this work started well before the emergence of COVID-19, some immediately-applicable examples emerge. Mapping the population allows overlay of other geographical data, e.g. location of residential care homes and general practices. This can support prioritisation of new models of care and service integration, such as utilising medical specialists in non-hospital settings [22]. As COVID-19-vulnerability data become available, this can be applied to the catchment, in order to develop preventive outreach options. These may include supporting virtual wards in care homes [23, 24] or utilising specialised acute diabetes teams to work with community care to support finding and keeping well the estimated 36,000 local people living with diagnosed and undiagnosed diabetes, a significant COVID-19 risk factor.

### Alcohol services

Understanding which local authority areas fall into which acute trust’s catchment has potential implications for the commissioning of public health services. Alcohol sales jumped 31.4% in March 2020 coinciding with the national COVID-19 ‘lockdown’ [25]. Although too early to tell if the medium to long-term impact results in increased alcohol-related harm, prior to the emergence of COVID-19, an estimated 80,000 (20%) adult residents in the catchment area were drinking in a way that is harmful to their health. Among inpatients, the prevalence rises to 30%, highlighting how hospitals are sentinel sites for identifying and addressing excess alcohol consumption [26].

Currently, however, substance-misuse services are commissioned by LAs: where services include hospital in-reach, only residents of the borough can usually be referred, leading to inequity of access. By defining the reach of the hospital catchment, it opens up the possibility of requesting constituent boroughs proportionally resourcing a comprehensive, hospital-based alcohol care team that would support all patients meeting clinical need criteria, and not determined by place of residence.

### Limitations

We believe this may be the first time an acute trust has generated such a health profile, and therefore we have no ready comparator against which to benchmark our findings. Nonetheless, there are some key limitations to consider when interpreting our work.

First, the PF method produces a geographically defined catchment population area which is not mutually exclusive. Because any LSOA meeting the defined activity proportion threshold is ‘claimed’ as being part of the catchment, in areas where there are multiple providers up to three hospitals could claim the same LSOA. This can result in some overlap of catchment areas and can be minimised by adjusting the threshold to optimise the tension between unclaimed LSOAs and too much overlap. This overlap should not, however, be considered problematic, as it reflects the underlying dynamism between healthcare providers and patients: people living in areas served by more than one hospital may vary where they access care whether through personal choice, or because hospitals offer different services.

There is no objective optimum that can satisfy the subjective preferences of researchers and policymakers in different clinical contexts where more or less overlap between catchments may be desired. We also acknowledge that the catchment population defined in this study is based on the intention of the study team to better understand the characteristics of the population of residents likely to attend CWFT and as such, different thresholds may be preferable for other Trusts of if used for other purposes.

Second, the model is underpinned by HES data which is primarily used for administrative purposes and concerns have been raised about its completeness and accuracy. Nonetheless it provides comprehensive coverage (>98% of hospital activity in England is NHS-funded), a requirement for our methodology, and is accepted as appropriate for health services research [27]. Further, our model makes use of high-level data (e.g. attendance type) which is likely less error prone than, for example, secondary diagnosis codes [28]. Further, the model is derived from one year of HES data. CWFT was formed by a merger of two hospitals in late 2015, so data prior to 2017/18 was not deemed to be reflective. Catchment areas are unlikely to be static over time, and further work should be undertaken as data becomes available to identify temporal trends.

HES provides retrospective data, and our existing model cannot project future service need. Integrating additional data sources (e.g. Quality and Outcomes Framework indicators), or obtaining permissions for HES linkage to other external datasets to build in health need predictions could be extremely valuable to aid service planning.

Third, there were challenges to overlaying public health datasets. Few indicators are available at small geographical level, and where available tend to be a number of years old (e.g. derived from the 2011 Census). More recent datasets are available but often at larger geographic scales with reduced granularity meaning an average rate was applied to smaller areas, potentially masking local variation.

In some instances, there are several datasets that could be used to examine a particular health need [29]. While sometimes there may be clear methodological arguments as to which dataset is more appropriate (e.g. self-reported smoking status may be more or less reliable when being shared directly with a clinician rather than via an anonymous survey), usually it is a pragmatic question. It is important that the resulting health need indicators are understood as estimates, and their limitations appropriately acknowledged, including that significance testing has not been conducted. Therefore, any initial indications of local health need may warrant further investigation.

## Conclusions

Each area of England is required to be part of an ICS in 2022, a body that will coordinate health and social care provision, and aim to improve health outcomes [30]. As part of this, it is anticipated that hospitals will be required to shift from fee-for-service to risk-sharing agreements. This will make it imperative that they understand their catchment population and its health needs, so they can define and manage this risk exposure. Yet such information is not routinely available: our work demonstrates the value a hospital-view of the local population could provide and opens up the question as to whether public health information should be made available nationally for every NHS hospital.

## Supplementary Information

**Additional file 1: Figure A1.** Pattern of assignment of LSOAs to CWFT and other providers along with overall catchment size for each proportional flow threshold. **Figure A2.** Percentage of patients within each LSOA assigned to the CWFT catchment at the 30% threshold attending CWFT. **Table A1.** Table showing Chelsea and Westminster NHS Foundation Trust (CWFT) catchment profile for: a) adult A&E, b) paediatric A&E and c) maternity population subgroups. **Table A2.** Summary of health indicators for which numerators and/or denominators other than catchment population size were used to derive a summary statistic.

## Data Availability

The data used in creating the catchment (Hospital Episode Statistics) are available to users meeting certain criteria on application to NHS Digital but restrictions apply to the availability of these data and so are not publicly available: https://digital.nhs.uk/data-and-information/data-tools-and-services/data-services/hospital-episode-statistics/users-uses-and-access-to-hospital-episode-statistics. Multiple publicly available data sets have been used in overlaying population estimates, with sources referenced in Table 2.
