# Supplementary material for: A picture of health: determining the core population served by an urban NHS hospital trust and understanding the key health needs
Source: BMC Public Health. 2022 Jan 12;22:75. doi: 10.1186/s12889-021-12373-5 (PMC8753015; doi:10.1186/s12889-021-12373-5)
Supplement: Supplementary file 1 — Additional file 1: Figure A1. Pattern of assignment of LSOAs to CWFT and other providers along with overall catchment size for each proportional flow threshold. Figure A2. Percentage of patients within each LSOA assigned to the CWFT catchment at the 30% threshold attending CWFT. Table A1. Table showing Chelsea and Westminster NHS Foundation Trust (CWFT) catchment profile for: a) adult A&E, b) paediatric A&E and c) maternity population subgroups. Table A2. Summary of health indicators for which numerators and/or denominators other than catchment population size were used to derive a summary statistic. [file 12889_2021_12373_MOESM1_ESM.docx]

**A Picture of Health: determining the core population served by an urban NHS hospital trust and understanding the key health needs**

**Appendix**

1. Thomas Beaney^1* #^
2. Jonathan M Clarke^2*^
3. Emily Grundy^3*^
4. Sophie Coronini-Cronberg^4, 5, 6^

*Joint first authors # Corresponding author

**Author affiliations**

1. Academic Clinical Fellow, *Department of Primary Care & Public Health, School of Public Health, Imperial College London, London, W6 8RP, UK.* ORCID ID: 0000-0001-9709-7264. Email: thomas.beaney@imperial.ac.uk
2. Sir Henry Wellcome Postdoctoral Fellow, Centre for Mathematics of Precision Healthcare, Department of Mathematics, Huxley Building, South Kensington Campus, Imperial College London, London SW7 2AZ, UK. ORCID ID: 0000-0003-1495-7746
3. Public Health Registrar, *Chelsea and Westminster Hospital NHS Foundation Trust, 369 Fulham Road, London, SW10 9NH, UK*
4. Consultant in Public Health, *Chelsea and Westminster Hospital NHS Foundation Trust, 369 Fulham Road, London, SW10 9NH, UK*
5. Honorary Senior Lecturer, *Department of Primary Care & Public Health, School of Public Health, Imperial College London, London, W6 8RP, UK*
6. Implementation Lead, *NIHR Applied Research Collaboration (ARC) North West London, 4^th^ Floor, Chelsea and Westminster Hospital, 369 Fulham Road, London, SW10 9NH, UK*

**Appendix**

**Figure A1:** Pattern of assignment of LSOAs to CWFT and other providers along with overall catchment size for each proportional flow threshold


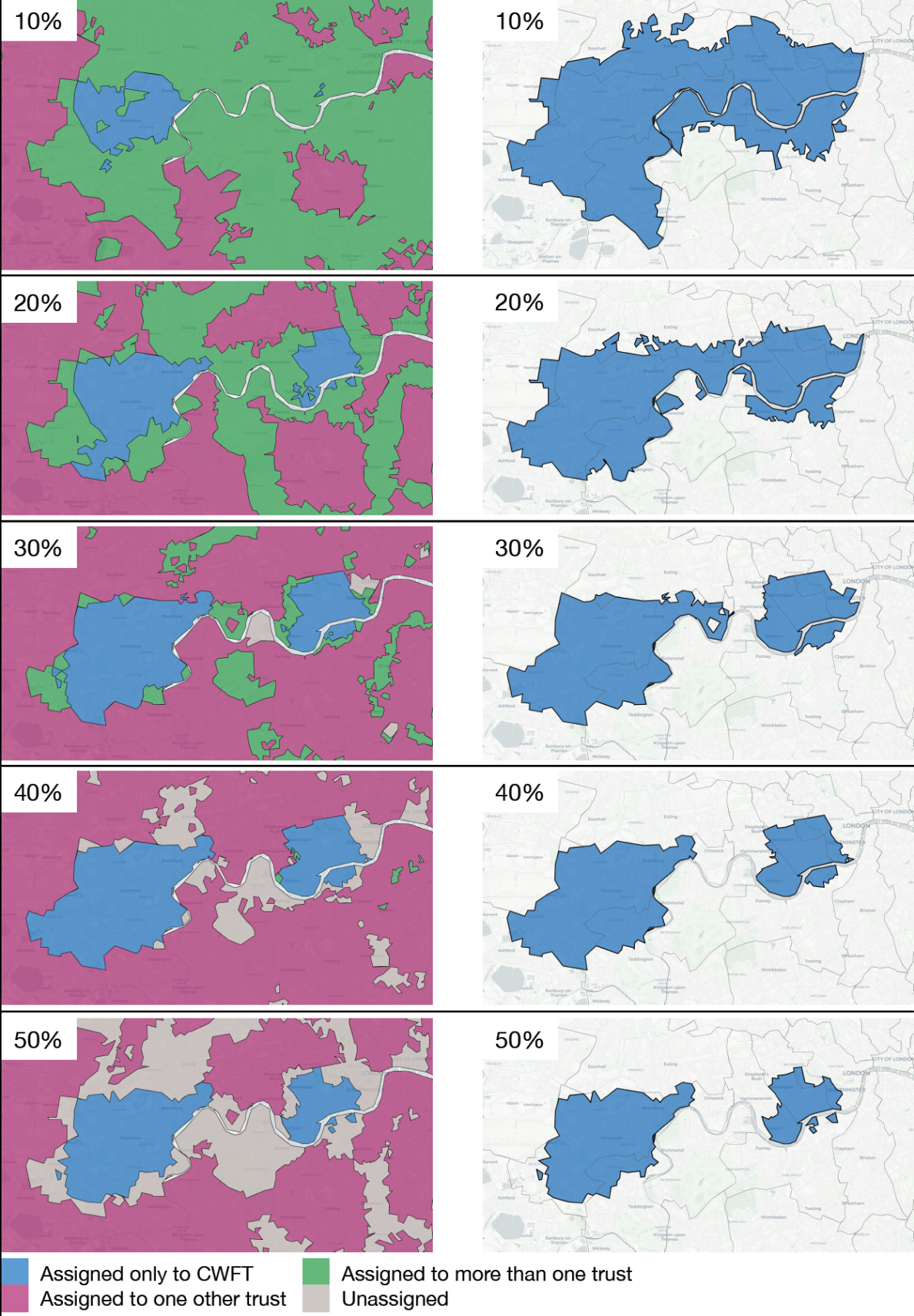


**Figure A2:** Percentage of patients within each LSOA assigned to the CWFT catchment at the 30% threshold attending CWFT.

**Table A1: Table showing Chelsea and Westminster NHS Foundation Trust (CWFT) catchment profile for: a) adult A&E, b) paediatric A&E and c) maternity population subgroups**

1. **Adult A&E**

|  | **Local Authority (LA)** | **STP region** | **LSOAs in CWFT catchment (N)** | **CWFT catchment population (N)** | **Proportion of CWFT catchment in LA (%)** | **Total LA population (N)** | **Proportion of LA population in CWFT catchment (%)** |
| --- | --- | --- | --- | --- | --- | --- | --- |
| **Adult A&E catchment population** | Ealing | NWL | 5 | 8,206 | 1.2% | 341,806 | 2.4% |
|  | Hammersmith and Fulham | NWL | 53 | 84,585 | 12.6% | 185,143 | 45.7% |
|  | Hillingdon | NWL | 1 | 1,944 | 0.3% | 306,870 | 0.6% |
|  | Hounslow | NWL | 135 | 257,341 | 38.3% | 271,523 | 94.8% |
|  | Kensington and Chelsea | NWL | 74 | 112,789 | 16.8% | 156,129 | 72.2% |
|  | Richmond-upon-Thames | SWL | 48 | 81,835 | 12.2% | 198,019 | 41.3% |
|  | Wandsworth | SWL | 50 | 92,808 | 13.8% | 329,677 | 28.2% |
|  | Westminster | NWL | 19 | 31,976 | 4.8% | 261,317 | 12.2% |
|  | **GRAND TOTAL** | **…** | **385** | **671,484** | **…** | **2,050,484** | **32.7%** |

1. **Paediatric A&E**

|  | **Local Authority (LA)** | **STP region** | **LSOAs in CWFT catchment (N)** | **CWFT catchment population (N)** | **Proportion of CWFT catchment in LA (%)** | **Total LA population (N)** | **Proportion of LA population in CWFT catchment (%)** |
| --- | --- | --- | --- | --- | --- | --- | --- |
| **Paediatric A&E catchment population** | Ealing | NWL | 118 | 202,215 | 19.1% | 341,806 | 59.2% |
|  | Hammersmith and Fulham | NWL | 112 | 181,653 | 17.2% | 185,143 | 98.1% |
|  | Hounslow | NWL | 142 | 269,100 | 25.5% | 271,523 | 99.1% |
|  | Kensington and Chelsea | NWL | 80 | 121,512 | 11.5% | 156,129 | 77.8% |
|  | Richmond-upon-Thames | SWL | 65 | 108,255 | 10.2% | 198,019 | 54.7% |
|  | Wandsworth | SWL | 71 | 130,256 | 12.3% | 329,677 | 39.5% |
|  | Westminster | NWL | 25 | 43,544 | 4.1% | 261,317 | 16.7% |
|  | **GRAND TOTAL** | **…** | **613** | **1,056,535** | **…** | **1,743,614** | **60.6%** |

1. **Maternity care**

|  | **Local Authority (LA)** | **STP region** | **LSOAs in CWFT catchment (N)** | **CWFT catchment population (N)** | **Proportion of CWFT catchment in LA (%)** | **Total LA population (N)** | **Proportion of LA population in CWFT catchment (%)** |
| --- | --- | --- | --- | --- | --- | --- | --- |
| **Maternity catchment population** | Ealing | NWL | 32 | 55,087 | 6.0% | 341,806 | 16.1% |
|  | Elmbridge | SH | 1 | 1,864 | 0.2% | 136,795 | 1.4% |
|  | Hammersmith and Fulham | NWL | 76 | 119,775 | 13.0% | 185,143 | 64.7% |
|  | Hillingdon | NWL | 1 | 1,944 | 0.2% | 306,870 | 0.6% |
|  | Hounslow | NWL | 139 | 264,272 | 28.7% | 271,523 | 97.3% |
|  | Kensington and Chelsea | NWL | 79 | 121,117 | 13.2% | 156,129 | 77.6% |
|  | Lambeth | SEL | 3 | 4,625 | 0.5% | 326,034 | 1.4% |
|  | Merton | SWL | 3 | 4,312 | 0.5% | 206,548 | 2.1% |
|  | Richmond-upon-Thames | SWL | 66 | 110,781 | 12.0% | 198,019 | 55.9% |
|  | Wandsworth | SWL | 98 | 177,174 | 19.3% | 329,677 | 53.7% |
|  | Westminster | NWL | 33 | 58,705 | 6.4% | 261,317 | 22.5% |
|  | **GRAND TOTAL** | **…** | **531** | **919,656** | **…** | **2,719,861** | **33.8%** |

LSOA = Lower Super Output Area; MSOA = Middle Super Output Layer; ICS=Integrated Care System; NWL=North West London; SWL=South West London.

LA total population estimates based on ONS Estimates of the population for the UK, England and Wales, Scotland and Northern Ireland: Mid-2019: April 2019 local authority district codes

**Equation applied to adjust for Index of Multiple Deprivation (IMD)**

Public health datasets for which national datasets broken down by IMD deciles were available were used to adjust relevant indicators at local authority or CCG level. This adjustment was made on the basis of the differential IMD profile of the constituent LSOAs within the CWFT catchment compared to each LA or CCG as a whole, using the following equation:

$$L^{'}= \frac{L \times\sum_{i=1}^{10} q_{i}r_{i}}{\sum_{i=1}^{10} p_{i}r_{i}}$$

- Where r_i_ is the normalised rate by IMD decile:

$$r_{i}= \frac{R_{i}}{R_{min}}$$

- Where R_i_ is the national rate for decile _i_ and R_min_ is the lowest rate in any one decile
- p_i_ is the proportion of LSOAs in decile i in the LA
- q_i_ is the proportion of LSOAS in decile i in the part of the LA in the catchment
- L is the value of the metric in the LA as a whole
- L′ is the value for the metric within the subset of the LA in the catchment

**Table A2: Summary of health indicators for which numerators and/or denominators other than catchment population size were used to derive a summary statistic.**

| **Indicator name** | **Numerator** | **Denominator** |
| --- | --- | --- |
| **Fuel Poverty** | Estimated number of households within the ‘all activity’ catchment area in 2017 experiencing fuel poverty | Estimated number of households within catchment area in 2017 |
| **Death attributable to poor air quality** | Number of deaths in 2018 within the ‘all activity’ catchment area which were attributed to poor air quality | Number of deaths in 2018 within catchment area |
| **Overcrowding** | Estimated number of households within the ‘all activity’ catchment area in 2011 experiencing overcrowding | Estimated number of households within catchment area in 2011 |
| **Life expectancy** | This dataset simply provides the average life expectancy/healthy life expectancy for each constituent MSOA within the ‘all activity’ catchment area. | |
| **Healthy life expectancy** | This dataset simply provides the average healthy life expectancy/healthy life expectancy for each constituent MSOA within the ‘all activity’ catchment area. | |
